# Recurrent Nausea and Hyponatraemia: Unmasking SIADH in a 50-Year-Old Woman

**DOI:** 10.1155/crie/1679940

**Published:** 2025-11-21

**Authors:** Chang Liu, Houliang Sun, Jingwu Zhao, Zhong Xin

**Affiliations:** ^1^Department of Endocrinology, Beijing Tongren Hospital, Capital Medical University, Beijing, China; ^2^Department of Neurology, Beijing Tongren Hospital, Capital Medical University, Beijing, China; ^3^Department of Neurosurgery, Beijing Tongren Hospital, Capital Medical University, Beijing, China

## Abstract

**Background:**

Syndrome of inappropriate antidiuretic hormone (SIADH) is a common cause of hyponatraemia that requires systematic investigation to identify underlying aetiologies. Central nervous system disorders represent an important but often underrecognised cause, particularly when patients present with non-specific gastrointestinal symptoms.

**Case Presentation:**

A 50-year-old woman presented with a 3-year history of intermittent nausea and vomiting that worsened over 20 days. Laboratory investigations revealed persistent hyponatraemia (serum sodium 125.4 mmol/L), low plasma osmolality (268.58 mOsm/kg H_2_O) and inappropriately elevated urine sodium (>40 mmol/L). Brain magnetic resonance imaging (MRI) demonstrated obstructive hydrocephalus with cerebellar tonsillar herniation, while magnetic resonance venography (MRV) showed stenosis of the right transverse sinus, sigmoid sinus and internal jugular vein.

**Management and Outcome:**

The patient was diagnosed with SIADH secondary to intracranial pathology. Treatment with fluid restriction (500–1000 mL/day) and oral salt supplementation (gradually increased to 2.5 g/day) successfully increased serum sodium to 130 mmol/L. Neurosurgical consultation recommended conservative management given the likely congenital developmental abnormalities. After 3 years of follow-up, the patient remains clinically stable with ongoing monitoring. Recent urine osmolality testing (611 mOsm/kg H_2_O) confirmed the SIADH diagnosis.

**Learning Points:**

This case demonstrates the importance of neuroimaging in unexplained SIADH and highlights practical diagnostic strategies when standard laboratory parameters like urine osmolality are unavailable. Central nervous system abnormalities should be systematically investigated in patients with persistent hyponatraemia and non-specific symptoms. Conservative management with fluid restriction and salt supplementation can be effective for mild-to-moderate SIADH cases.

## 1. Introduction

Syndrome of inappropriate antidiuretic hormone (SIADH) is a common cause of hyponatraemia and requires systematic investigation of underlying causes. Central nervous system disorders represent an important but often underrecognised cause of SIADH, particularly when patients present with non-specific gastrointestinal symptoms. Diagnosis traditionally requires comprehensive biochemical testing, including urine osmolality measurement, which may be unavailable in many clinical settings. This case demonstrates the diagnostic approach to persistent hyponatraemia in a patient with chronic nausea and vomiting, ultimately revealing central nervous system disorders as the underlying cause. It illustrates practical strategies for SIADH diagnosis when standard laboratory parameters are unavailable and emphasises the importance of neuroimaging in unexplained cases, providing valuable insights for clinicians managing similar presentations.

## 2. Case Presentation

A 50-year-old woman presented with a 3-year history of intermittent nausea and vomiting, which had worsened over the past 20 days. She denied abdominal pain, diarrhoea, dizziness, headache and palpitations. Despite experiencing these symptoms for 3 years, her previous medical care was limited to symptomatic treatment only. During prior doctor visits, she received treatments that temporarily alleviated her symptoms, but each time they would eventually return. Limited investigations were performed to identify the underlying cause, but she had never undergone electrolyte testing before this presentation.

Gastroscopy performed during an outpatient visit 20 days prior to admission showed chronic atrophic gastritis, reflux oesophagitis and hiatal hernia (25 June 2022). Recent outpatient tests revealed serum sodium 125.3 mmol/L (14 July 2022, reference range 137–147 mmol/L). After 1 week of oral salt supplementation (sodium chloride capsules, initially one capsule three times daily, ~1.3 g/day) and rabeprazole treatment, her symptoms slightly improved, but serum sodium remained low (128.7 mmol/L, 21 July 2022). Initial symptomatic treatment was initiated without establishing an aetiological diagnosis. She was admitted for further investigation.

Her medical history included mild, intermittent hypertension managed without pharmacological therapy. There was no relevant drug history or family history of genetic disorders.

On examination, the patient was alert and oriented. During hospitalisation, the patient's heart rate ranged from 70 to 75 beats/min, and supine blood pressure ranged from 128/75–145/86 mmHg. There was no lymphadenopathy. Chest and cardiovascular examinations were unremarkable. The abdomen was soft and non-tender, with no evidence of organomegaly. No peripheral oedema was noted. No significant visual field defects or decreased visual acuity were found.

### 2.1. Investigations

• Serum sodium: 125.4 mmol/L (5 August 2022).
• Plasma osmolality: 268.58 mOsm/kg H_2_O (reference range 280–295 mOsm/kg H_2_O, 5 August 2022).
• Normal blood glucose, liver and renal function, lipids, and BNP (B-type natriuretic peptide, 5 August 2022).
• Normal pituitary hormones, thyroid function, cortisol, ACTH (Adrenocorticotropic Hormone), tumour markers and immunological tests. (5 August 2022–8 August 2022).
• Urine sodium: >40 mmol/L (multiple measurements [9 August 2022 and 10 August 2022] [Reference range: typically <20 mmol/L in euvolemic hyponatremia]) [1].

As part of the systematic investigation to identify the underlying cause of hyponatraemia, brain imaging was performed. Magnetic resonance imaging (MRI) and magnetic resonance venography (MRV) were indicated because SIADH can be caused by central nervous system disorders affecting the hypothalamus–pituitary axis, and the patient's persistent symptoms suggested a possible neurological component.

• Brain MRI: Supratentorial obstructive hydrocephalus, cerebellar tonsillar herniation, compressed fourth ventricle, periventricular demyelination (11 August 2022).
• Brain MRV: Stenosis of the right transverse sinus, right sigmoid sinus and internal jugular vein; hydrocephalus (18 August 2022).

Treatment with fluid restriction to ~500–1000 mL per day and oral salt supplementation (sodium chloride capsules gradually increased from one capsule three times daily [~1.3 g/day] to two capsules three times daily [~2.5 g/day]) increased serum sodium to 130 mmol/L (Table 1).

### 2.2. Questions and Answers

#### 2.2.1. Q1. What Do the Blood Tests Show?

The blood tests show:

Electrolyte abnormalities:

• Persistent hyponatraemia with serum sodium of 125.4 mmol/L (previously 125.3 mmol/L in outpatient testing, improving slightly to 128.7 mmol/L after initial treatment).
• Urine sodium persistently elevated at >40 mmol/L despite hyponatraemia (measured multiple times).
• Osmolality: Decreased plasma osmolality at 268.58 mOsm/kg H_2_O.

The patient exhibited normal findings across multiple systems, including normal blood glucose, liver and renal function tests, lipid profile, BNP, pituitary hormones, thyroid function, cortisol, ACTH, tumour markers and immunological tests.

The pattern of laboratory findings (hyponatraemia with low plasma osmolality and inappropriately high urine sodium) in a euvolaemic patient is consistent with SIADH.

#### 2.2.2. Q2. What is the Diagnosis?

Based on the clinical presentation and laboratory findings in the case, the diagnosis is SIADH, secondary to intracranial pathology (obstructive hydrocephalus and venous sinus stenosis) [2].

Diagnosis of SIADH is supported by:

• Persistent hyponatraemia (serum sodium 125.4 mmol/L).
• Low plasma osmolality (268.58 mOsm/kg H_2_O).
• Inappropriately high urine sodium (>40 mmol/L on multiple measurements) despite hyponatraemia.
• Normal renal, liver, and thyroid function tests.
• Absence of oedema or signs of volume depletion (euvolemic state).
• Improvement with fluid restriction and salt supplementation.

SIADH is a clinical and biochemical syndrome characterised by hypotonic hyponatraemia, concentrated urine and normal blood volume. It is primarily caused by excessive release of arginine vasopressin (AVP), which impairs the body's ability to excrete free water [2]. Neurological symptoms such as nausea, vomiting, headache and confusion are common in SIADH and are directly related to the severity of hyponatraemia [3].

SIADH aetiology includes: malignancies (small cell lung cancer and others) with direct AVP secretion by tumour cells [4, 5], central nervous system disorders (cerebral infections, trauma, hydrocephalus) disrupting hypothalamic–pituitary function [6], pulmonary diseases affecting osmoreceptors or triggering AVP release through inflammatory pathways [7], medications (SSRIs [selective serotonin reuptake inhibitors], carbamazepine) [8] and genetic disorders [9]. In this case, hydrocephalus and intracranial abnormalities were identified as the underlying cause.

Bartter and Schwartz describe the criteria for the diagnosis of SIADH as decreased serum osmolality (<275 mOsm/kg H_2_O), increased urine osmolality (>100 mOsm/kg H_2_O), euvolaemia, increased urine sodium (>20 mmol/L), and no other cause for hyponatraemia [1]. In this case, SIADH was diagnosed by excluding pseudohyponatraemia (hyperglycaemia, hyperlipidaemia), hypovolaemic hyponatraemia (vomiting, diarrhoea), hypervolaemic hyponatraemia (heart failure, cirrhosis) and other euvolaemic conditions (primary polydipsia, adrenal insufficiency). An important differential diagnosis in patients with intracranial pathology is cerebral salt wasting (CSW), which was excluded in this case based on several key features. CSW typically occurs within 1–2 weeks following acute neurosurgical procedures, traumatic brain injury or subarachnoid haemorrhage, and is characterised by hypovolaemia preceding hyponatraemia due to inappropriate renal sodium and fluid loss. In contrast, our patient presented with chronic symptoms without recent neurosurgical intervention and demonstrated euvolaemia. Importantly, the patient responded appropriately to fluid restriction with improvement in hyponatraemia—a response that would have induced volume depletion in CSW patients. Additionally, CSW is rare even in neurosurgical settings, accounting for only 3.7% of hyponatraemia cases [10]. Although urine osmolality testing was not available at the time of initial diagnosis, the diagnosis was supported by euvolaemic hyponatraemia, elevated urine sodium, clinical presentation, negative investigations and an excellent response to fluid restriction.

#### 2.2.3. How Should This Condition be Managed?

Management of SIADH involves treating both the underlying cause and correcting hyponatraemia. The approach is based on established clinical guidelines, including the European Clinical Practice Guideline on diagnosis and treatment of hyponatraemia [3]. The following steps are recommended:

1. Fluid restriction:
  • This is the first-line treatment for mild to moderate hyponatraemia (serum sodium 125–135 mmol/L). Daily fluid intake should be restricted to 500 mL less than daily urine volume [10] or 500 mL/day adjusted according to the serum sodium levels [11].
2. Oral salt supplementation:
  • Oral sodium chloride supplementation helps to counteract inappropriate renal sodium excretion, thereby increasing serum sodium concentration.
3. Hypertonic saline:
  • For severely symptomatic hyponatraemia (severe neurological symptoms, usually serum sodium <125 mmol/L), prompt intravenous infusion of 3% hypertonic saline (150 mL over 20 min, potentially repeated up to twice after checking serum sodium) is recommended to achieve a target increase of 5 mmol/L in serum sodium concentration, with close monitoring to avoid overcorrection, which can lead to central pontine myelinolysis [3].
4. Vasopressin receptor antagonists:
  • Drugs such as tolvaptan are increasingly used to treat SIADH by blocking AVP receptors [12].
5. Monitoring and follow-up:
  • Regular monitoring of serum sodium levels and neurological status is essential.
  • Repeat imaging may be necessary to assess the progression of intracranial abnormalities.
6. Treatment of underlying cause
  • Addressing the primary aetiology is essential. In this case, neurosurgical consultation for management of obstructive hydrocephalus should be considered.

In this case, fluid restriction and oral salt supplementation successfully increased serum sodium to 130 mmol/L (18 August 2022).

### 2.3. Outcome and Follow-up

The neurosurgeon considered that the findings most likely represented congenital developmental abnormalities and recommended conservative management rather than immediate surgical intervention given the patient's stable clinical condition, successful SIADH management, and the potential surgical risks. After nearly 3 years of careful observation, the patient remains clinically stable with ongoing monitoring of both intracranial pathology and serum sodium levels. Recent follow-up tests in another hospital revealed a urine osmolality of 611 mOsm/kgH_2_O confirming the SIADH diagnosis (20 August 2025).

### 2.4. Learning Points

• SIADH is a common cause of hyponatraemia and requires systematic investigation of underlying causes.
• Brain imaging is essential in cases of unexplained SIADH, as intracranial abnormalities are a frequent cause.
• Treatment should focus on both correcting hyponatraemia and addressing the underlying condition.
• Regular monitoring is crucial to prevent complications, such as overcorrection of sodium levels.

## Figures and Tables

**Table 1:** Serial electrolyte measurements overtime.

Electrolyte measurements	14 July 2022	21 July 2022	5 August 2022	7 August 2022	13 August 2022	16 August 2022	18 August 2022	24 August 2022	Reference range
Sodium (Na) (mmol/L)	125.3	128.7	125.4	129.4	128.7	128.0	130.4	131.2	137–147
Potassium (K) (mmol/L)	4.42	4.97	4.24	4.48	3.98	3.7	3.7	5.1	3.5–5.3
Chloride (Cl) (mmol/L)	93.5	96.4	94.2	95.5	95.9	95.7	95.2	98.7	99–110

## Data Availability

The data that support the findings of this study are available upon request from the corresponding author. The data are not publicly available due to privacy or ethical restrictions.
